# Chronic pain conditions and risk of suicidal behavior: a 10-year longitudinal co-twin control study

**DOI:** 10.1186/s12916-022-02703-8

**Published:** 2023-01-05

**Authors:** C. Chen, E. Pettersson, A. G. Summit, K. Boersma, Z. Chang, R. Kuja-Halkola, P. Lichtenstein, P. D. Quinn

**Affiliations:** 1 Department of Medical Epidemiology and Biostatistics, Karolinska Institutet, Box 281, 17177 Stockholm, Sweden; 2 Department of Applied Health Science, School of Public Health, Indiana University, Bloomington, IN USA; 3 Center for Health and Medical Psychology (CHAMP), School of Law, Psychology and Social Work, Örebro University, Örebro, Sweden

**Keywords:** Chronic pain comorbidity, General factor of pain, Suicidal behaviors, Co-twin control design, Longitudinal study, Suicide prevention

## Abstract

**Background:**

Understanding the relationship between chronic pain conditions and suicidal behavior—suicide attempt, other intentional self-harm, and death by suicide—is imperative for suicide prevention efforts. Although chronic pain conditions are associated with suicidal behaviors, these associations might be attributed to unmeasured confounding or mediated via pain comorbidity.

**Methods:**

We linked a population-based Swedish twin study (*N*=17,148 twins) with 10 years of longitudinal, nationwide records of suicidal behavior from health and mortality registers through 2016. To investigate whether pain comorbidity versus specific pain conditions were more important for later suicidal behavior, we modeled a general factor of pain and two independent specific pain factors (measuring pain-related somatic symptoms and neck-shoulder pain, respectively) based on 9 self-reported chronic pain conditions. To examine whether the pain-suicidal behavior associations were attributable to familial confounding, we applied a co-twin control model.

**Results:**

Individuals scoring one standard deviation above the mean on the general pain factor had a 51% higher risk of experiencing suicidal behavior (odds ratio (OR), 1.51; 95% confidence interval (CI), 1.34–1.72). The specific factor of somatic pain was also associated with increased risk for suicidal behavior (OR, 1.80; 95% CI, 1.45–2.22]). However, after adjustment for familial confounding, the associations were greatly attenuated and not statistically significant within monozygotic twin pairs (general pain factor OR, 0.89; 95% CI, 0.59–1.33; somatic pain factor OR, 1.02; 95% CI, 0.49–2.11)

**Conclusion:**

Clinicians might benefit from measuring not only specific types of pain, but also pain comorbidity; however, treating pain might not necessarily reduce future suicidal behavior, as the associations appeared attributable to familial confounding.

**Supplementary Information:**

The online version contains supplementary material available at 10.1186/s12916-022-02703-8.

## Background

Globally, 9 people per 100,000 died from suicide in 2019 [1]. In Sweden, suicide is one of the most common single causes of death. With a population of 10 million people, approximately 1500 individuals die by suicide every year [2]. Although suicide is only the 10th leading cause of death in the USA, it affects younger and middle-aged individuals disproportionally. In particular, it is the 2nd leading cause of death for people ages 10–34 and the 4th leading cause among people ages 35–44. Furthermore, suicidal ideation and attempts are even more prevalent, as 12 million Americans seriously considered suicide in 2019, 3.5 million planned a suicide attempt, and 1.4 million attempted suicide [3].

Suicide definitions provided by the US Centers for Disease Control and Prevention include suicidal self-directed violence (death by suicide, suicide attempt, suicidal ideation) and self-directed violence with undetermined or nonsuicidal intent [4]. Suicidal behaviors, including suicide attempts, other intentional self-harm, and death by suicide, are often viewed as existing across a gradient, such that there is a progression in suicidality severity over time are often viewed as existing across a gradient, such that there is a progression in suicidality severity over time [5, 6]. This suggests that early identification of predictors of suicidal behaviors is of paramount importance. One important predictor is chronic pain, which is associated with both suicidal behaviors and death by suicide [7–10].

Chronic pain is a serious, long-standing, and debilitating condition affecting approximately 1 person out of 5 worldwide [11–13]. It is associated with a heavier economic burden, poorer quality of life, worse mental well-being, and decreased physical functioning [14–19]. Based on these findings, several clinical guidelines now highlight the importance of considering chronic pain as a potential risk factor for suicidality [20–23]. Likewise, the World Health Organization (WHO) acknowledges chronic pain as an individual key risk factor for suicide [24].

Consumption of higher doses of prescribed opioids in pain management has been linked to increased risk of death by suicide [25], and in the context of the ongoing epidemic of opioid-related mortality, the close ties among pain, opioids, and suicide risk [26, 27] suggest that it is imperative for prevention efforts to consider the relation between pain and suicide. In fact, neurobiological opioid systems are likely involved in chronic pain and many of its evidence-based treatments and also play an important role in suicidal behaviors that were previously attributed solely to the pituitary–hypothalamic–adrenal axis [28, 29]. Nevertheless, research on the causal role of pain in suicide has been hampered by two important limitations.

First, a challenge remains in that chronic pain conditions often coexist [30–32]. For example, there are positive associations among quite different types of pain, including irritable bowel syndrome (IBS), myalgic encephalomyelitis/chronic fatigue syndrome (CFS), interstitial cystitis/painful bladder syndrome, chronic tension-type headache, migraine headache, chronic lower back pain, temporomandibular disorder (TMD), fibromyalgia (FM), endometriosis, and vulvodynia [33]. Twin and molecular genetic research have shown that this co-existence appears largely genetic in origin [34–36]. Because of this extensive pain comorbidity, it remains unclear whether different types of pain conditions increase risk of later suicide uniquely, or whether they do so in combination. A next step is therefore to try to understand whether associations with increased risk of suicide are specific to any particular pain condition or pain comorbidity more broadly [37–39]. In the present study, we addressed this by explicitly modeling overlapping pain conditions via a general factor model, which allows for decomposing their overlap into one general and several specific components [40]. We then examined associations between the general and the specific factors with later suicidal behavior, thereby generating an estimate of how much of the associations that can be attributed to pain comorbidity.

Second, past research examining the chronic pain-suicidality link has failed to control for unmeasured confounding. Studies have demonstrated that not only does chronic pain have a partly genetic origin but so does suicidal behavior [35, 41, 42]. Therefore, the associations between chronic pain conditions and suicidal behavior could potentially be confounded by unmeasured genetic factors [43]. Understanding the nature (causal vs. confounding) of the association between chronic pain and suicidal behavior is necessary to guide effective suicide prevention among people with chronic pain conditions. If the association were wholly attributable to confounding, then treating pain would not be expected to lower risk of later suicide. To examine this issue, we employed a co-twin control design to investigate to what extent the observed associations remained after controlling unmeasured familial confounding shared within twin pairs [44].

## Methods

### Sample

During 2005 and 2006, all twins born in Sweden 1958–1985 (42,582 twin individuals aged 20–47 years at assessment) were contacted to participate in a study about physical and mental health (the Study of Twin Adults: Genes and Environment; STAGE). About 60% (25,418 individuals) of all eligible twins responded to a web- or phone-based survey assessing a wide variety of common complex health problems as well as related exposures [45]. STAGE was approved by the Stockholm Regional Ethics Committee with reference number 2010-322-31/1 and all participants gave informed consent. The study has been described in detail elsewhere [45, 46]. After excluding twins who had uncertain zygosity (787 individuals) or who had entirely missing data for themselves or their co-twins (7483 individuals), we analyzed a sample of 17,148 individuals (57% female), including 7126 monozygotic (MZ) twins and 10,022 dizygotic (DZ) twins.

### Measures

#### Exposures

In STAGE, the twins self-reported on a range of painful symptoms and indicators using scales that have been reported and validated previously. We included 9 scales that captured a broad range of pain conditions (chronic widespread pain, irritable bowel syndrome, headache (including migraine), neck pain, shoulder pain, lower back pain, joint pain, bladder pain, and chronic fatigue syndrome; see Additional file 1: Supplemental Method [30, 34, 47–57] for details regarding the items and scales). We modeled each condition as a symptom or severity count (see Table 1 for descriptives). Chronic widespread pain, the cardinal symptom of fibromyalgia, is prevalent and co-occurs with numerous symptom-based conditions such as chronic fatigue syndrome, joint pain, headache, and irritable bowel syndrome [30]. Low back pain, in particular, is the leading cause of disability worldwide [58]. Bladder pain is a heterogeneous condition involving chronic and often severe pain perceived in the urinary bladder [59]. Additional file 1: Table S1 showed how chronic pain conditions look by MZ/DZ status.

**Table 1:** Frequency distribution table of the nine pain conditions and suicidal behaviors

Measures	***n***	% (***n***/17,148)
Chronic widespread pain		
0	11,669	68.0
1	915	5.3
2	100	0.6
3	161	0.9
4	526	3.1
Missing	3777	22.0
Joint pain		
0	14,312	83.5
1	974	5.7
Missing	1862	10.9
Lower back pain		
0	8693	50.7
1	876	5.1
2	892	5.2
3	1149	6.7
Missing	5538	32.3
Neck pain		
0	8831	51.5
1	931	5.4
2	929	5.4
3	945	5.5
Missing	5512	32.1
Shoulder pain		
0	9302	54.2
1	706	4.1
2	773	4.5
3	855	5.0
Missing	5512	32.1
Irritable bowel syndrome		
0	13,944	81.3
1	493	2.9
2	428	2.5
3	308	1.8
4	362	2.1
5	243	1.4
6	71	0.4
Missing	1299	7.6
Headache		
0	4890	28.5
1	5675	33.1
2	4854	28.3
Missing	1729	10.1
Bladder pain		
0	11,257	65.6
1	2346	13.7
2	523	3.0
3	335	2.0
4	102	0.6
Missing	2585	15.1
Chronic fatigue syndrome		
0	11,363	66.3
1	1224	7.1
2	1568	9.1
3	1205	7.0
Missing	1788	10.4
Suicidal behaviors		
0	16,878	98.4
1 (suicidal attempt)	231	1.3
2 (injuries of undetermined intent only )	13	0.1
3 (death by suicide)	26	0.2

#### Outcomes

Via personal identity numbers, the Swedish Twin Registry linked the twin participants to records from the National Patient Register [60] (hospitalizations in 1997–2016 and visits to psychiatrists or other outpatient medical specialists in 2001–2016) and the Cause of Death Register [61] (deaths through 2016). We thus assessed suicidal behavior by identifying those who received any clinical diagnoses of intentional self-harm (including suicide attempt) or injuries of undetermined intent in inpatient hospitalizations or visits to medical specialists, as well as those who died by suicide (*ICD-10* codes: X60-X84, Y10-Y34, Y87.0, Y87.2; see Table 1 for descriptives). The register data covered all individuals residing in Sweden, and the follow-up was 10 years (i.e., until 2016) after the STAGE assessment (in addition to prior suicidal behavior). Like prior register-based studies, we included injuries of undetermined intent to also capture possible suicidal behavior [62]. The prevalence of suicidal behaviors in this study is 1.6% (1.58% for DZ twins, 1.57% for MZ twins). Additional file 1: Table S2 showed how chronic pain conditions look by suicidal behaviors.

#### Covariates

Sex, age, and cancer (identified from National Patient Register) were included as covariates. Cancer-related pain generally occurs as a result of perioperative procedures, nerve damage caused by radiation or chemotherapy treatments, or mucositis and therefore may be qualitatively distinct from the pain conditions of interest [63]. Thus, we wanted to account for cancer-related pain.

### Statistical analyses

First, we conducted logistic regression to examine the extent to which each pain condition was associated with risk of suicidal behavior both prior to and after the STAGE assessment date (i.e., from 1997 through 2016) by regressing suicidal behavior on each pain scale separately. We reported standardized results, such that odds ratios represent the odds of suicidal behavior associated with a 1-standard-deviation change in a pain scale or factor for all our analyses.

Second, we regressed suicidal behavior on all nine pain scales simultaneously in a multiple regression to examine whether any pain condition was particularly associated with outcome, above and beyond what it shared with the other pain scales.

Third, whereas the multiple regression model estimates unique associations, it does not provide an estimate for how the shared variance among the predictors is associated with the outcome. Therefore, to formally model how pain comorbidity was associated with later suicidal behavior, we fitted a bifactor model to the pain scales. A bifactor model includes both a general and several specific factors. The general factor captures covariation shared among all pain scales, whereas the specific factors capture covariation unique to subsets of pain scales over and above the general factor. This separation between shared and specific variation is achieved by constraining the correlation between the general and specific factors at zero (however, we estimated correlations among the specific latent trait factors).

The development of the bifactor model was a two-step procedure: In the first step, we fit an exploratory factor analysis (EFA) to the pain conditions because we did not have strong hypotheses for how the pain scales might covary. In EFA, all items are allowed to load (i.e., cross-load) on all factors. We examined the Eigenvalues to determine how many factors to extract, and the first five were 4.07, 1.04, 0.80, 0.75, and 0.67. Although this indicated a two-factor solution, we extracted three exploratory factors to allow for capturing as much specific variance as possible. We then rotated the three-factor EFA toward a bifactor solution.

In the second step, we re-fit the EFA as a confirmatory factor analysis (CFA), in which we forced the EFA-specific factors loading less than |0.30| to load at 0.0. We did this to ensure that any ensuing specific factor-suicidal behavior associations could not be attributed to cross-loadings (because these were constrained to zero). Because three scales (wide, back, and joint pain) loaded less than |0.30| on all specific factors, we employed a so-called CFA bifactor-(S-1) approach, whereby a set of indicators serve as a reference group for the general factor [64]. In other words, those three pain scales loaded only on the general factor and did not form a specific factor (Additional file 1: Fig. S1). This way, the general factor is readily interpretable as capturing wide, back, and joint pain (along with their pain comorbidities), while also avoiding potentially anomalous specific factor loading patterns.

The CFA general factor model fitted the data well (root mean square error of approximation [RMSEA] = 0.011; confirmatory fit index [CFI] = 0.997; Tucker-Lewis index [TLI] = 0.997; ${\upchi}_{36}^2$=24780.003; *P* < .001). The scale capturing widespread pain loaded close to unity on the general factor, and the rest of the pain scales also loaded substantially on it (standardized mean loading = 0.58; range 0.32, 0.99). Therefore, the general factor could be interpreted as capturing widespread pain and all its pain correlates. The first specific factor captured somatic symptoms (chronic fatigue syndrome loaded 0.33; irritable bowel syndrome loaded 0.49; headache loaded 0.33; and bladder pain loaded 0.37), and the second specific factor captured neck-shoulder pain (neck pain loaded 0.71, shoulder pain loaded 0.34) (Additional file 1: Table S3).

We then regressed suicidal behavior on this CFA model within a structural equation modeling (SEM) framework, with sex, age and cancer included as covariates. An advantage of working within an SEM framework is that the latent pain factors are estimated as having perfect reliability, such that the ensuing associations with suicidal behavior were not attenuated by measurement error. To account for the non-independence of twin data, family clusters were specified, and standard errors were estimated using a cluster robust sandwich estimator.

Fourth, we assumed that part of the association between pain conditions and later suicide could be attributed to unmeasured confounding factors shared by twins within pairs (e.g., family socio-economic status, childhood conditions, genetics). We therefore estimated the pain-suicide associations within twin pairs to control for such factors using Allison’s hybrid (or co-twin control) approach [65]. Technically, the hybrid approach involves estimating a latent twin-pair intercept for the outcome and then allowing this pair intercept to correlate with all exposures. Conceptually, this is akin to including a covariate in the regression model to estimate unique exposure-outcome association among those with similar scores on the covariate, except that the covariate is a latent variable which has the same value for both twins in a pair. In DZ twins, the pair intercept captures 50% of the segregating genes and shared environmental childhood conditions, and in MZ twins the pair intercept instead captures 100% of the segregating genes. Thus, to the extent the observed associations attenuate more among MZ compared to DZ twins, this indicates the possible presence of genetic confounding [66].

### Sensitivity analysis

First, to rule out reverse causation we repeated the univariable and multiple regression analyses excluding participants with suicidal behavior diagnoses before the STAGE survey. Second, to examine whether the associations persisted with a stricter outcome classification, we excluded the “injuries of undetermined intent” diagnoses (*ICD-10* codes: X60-X84, Y87.0). Third, some participants died by non-suicide causes during follow-up. We therefore conducted a Cox model with censoring by death due to other causes. Fourth, to examine the extent to which associations were explained by underlying somatic conditions, we included self-reported somatic disorders (chronic bronchitis, Crohn's disease, diabetes, glandular disease (including everything but goiter), irregular cardiac rhythm/atrial fibrillation, liver disease (for example, cirrhosis), multiple sclerosis, goiter, ulcerative colitis, recurring urinary tract problems, intestinal (duodenal) ulcer, stomach ulcer) as covariates in the univariable and multiple regression analyses.

## Results

### Univariable and multiple logistic regression model

**Fig. 1:**
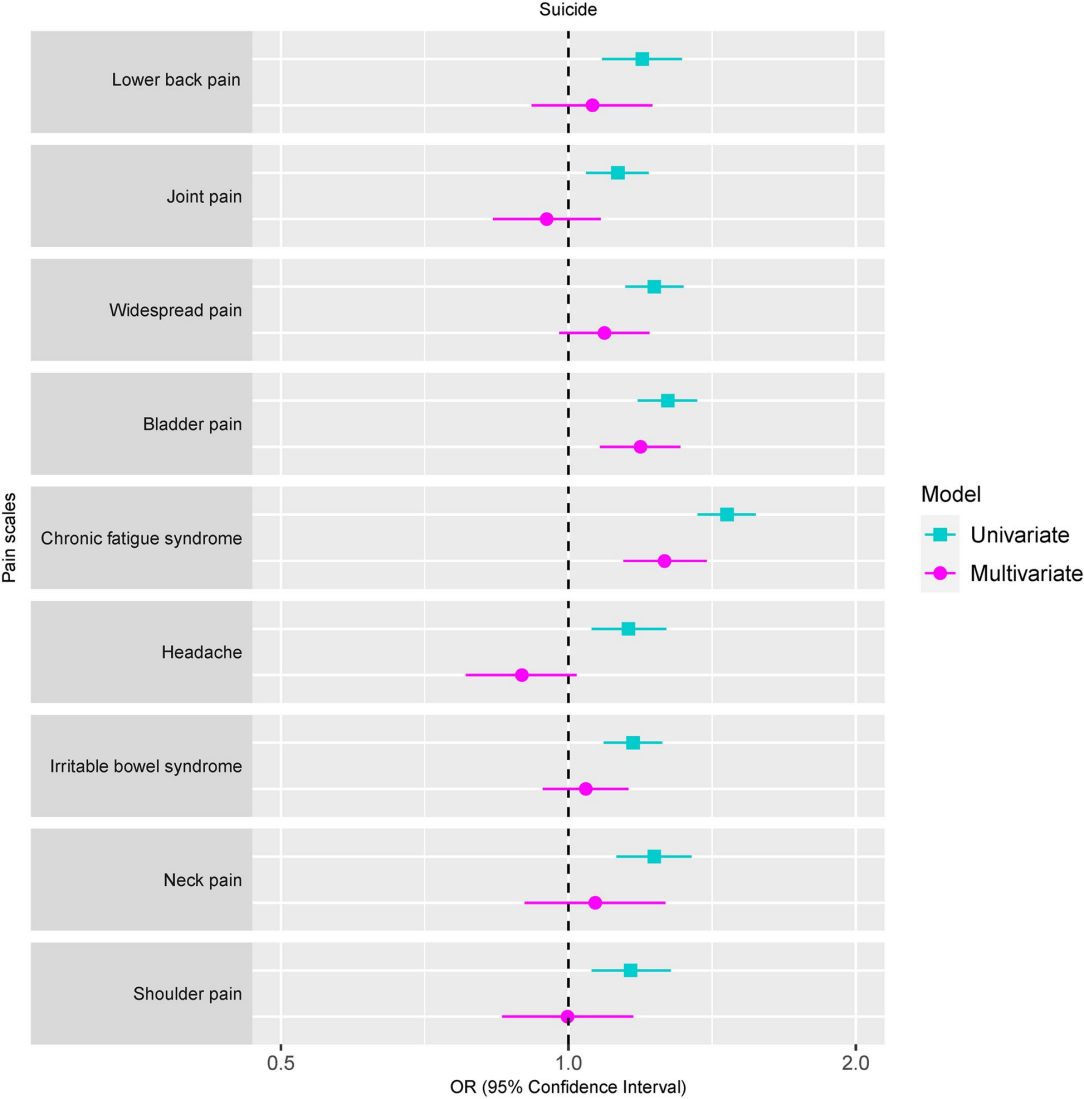
Associations between pain conditions and suicidal behaviors using univariate and multivariate logistic regression model. Note: Models were adjusted for age, sex, and cancer

Individuals who scored 1 SD above the mean on the pain conditions had significantly higher risk of later suicidal behavior when estimated separately (OR ranged from 1.13 to 1.47). However, after including all the pain conditions in one multiple regression model, only two pain conditions (chronic fatigue syndrome OR=1.26, 95% CI [1.14, 1.40]; bladder pain OR=1.19, 95% CI [1.08, 1.31]) remained uniquely associated with suicidal behavior (Fig. 1). This attenuation suggests that broad pain comorbidity might account for a sizeable part of the associations.

### Suicide behavior regressed on latent general factor and specific factors

#### Individual level associations

**Fig. 2:**
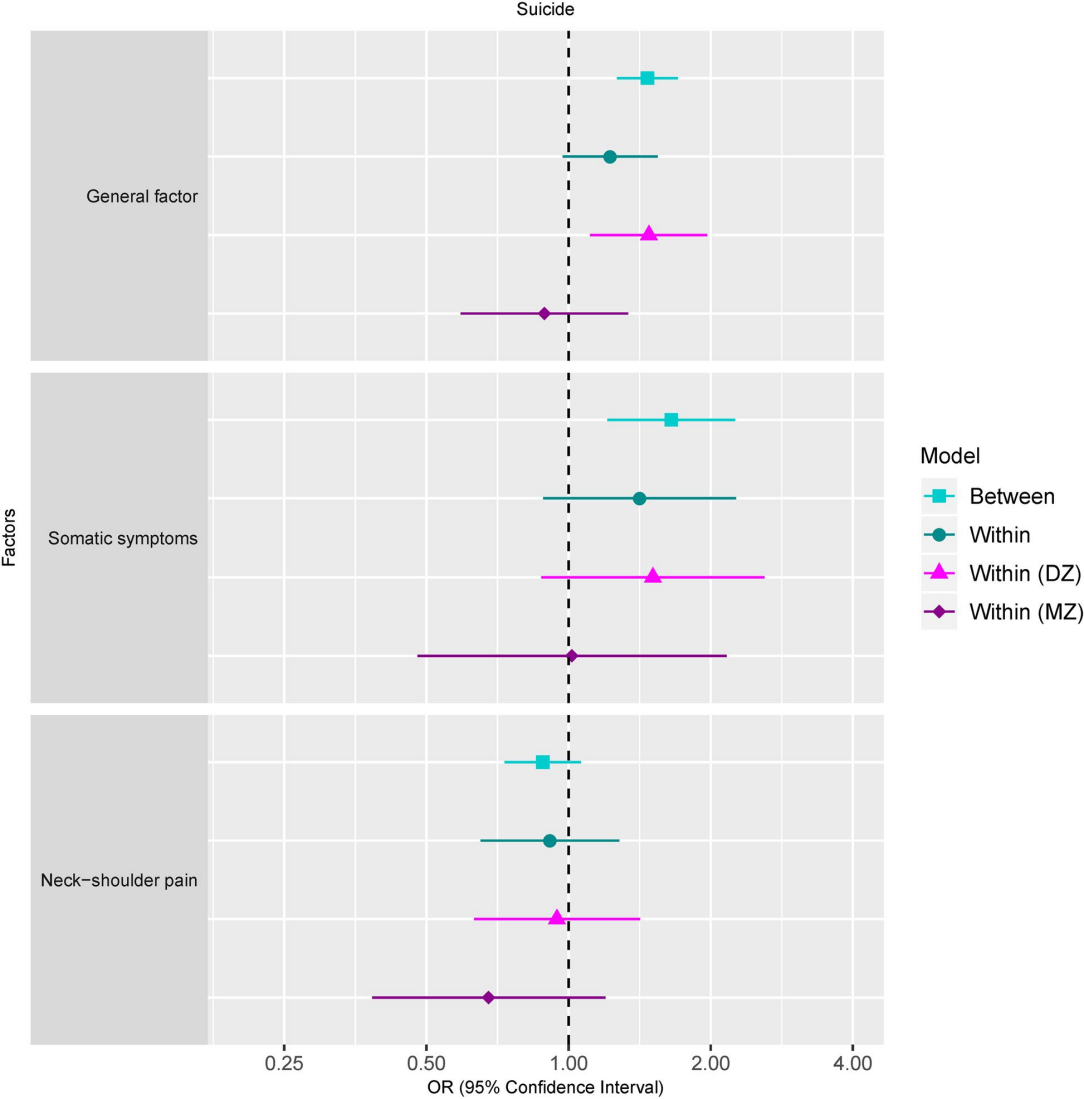
Associations between latent pain factors and suicidal behaviors between all twin pairs (model: between), within all twin pairs (model: within), within monozygotic twin pairs (model: within (MZ)), and within dizygotic twin pairs (model: within (DZ)). Note: Models were adjusted for age, sex, and cancer

As displayed in Fig. 2, individuals scoring one standard deviation above the mean on the general factor had 51% higher odds of suffering later suicidal behavior (OR=1.51, 95% CI [1.34, 1.72]). With regard to the specific factors, the somatic symptoms factor was also statistically significantly associated with suicidal behavior (OR=1.80, 95% CI [1.45,2.22]), whereas the neck-shoulder pain factor was not (OR=0.90, 95% CI [0.83,1.08]).

#### Within-pair associations (co-twin control associations)

After controlling for unmeasured between-pair confounding, the association between the general factor of pain and suicidal behavior attenuated when all twins (regardless of zygosity) were combined (OR=1.21, 95% CI [0.97, 1.52]). Although this association was statistically significantly different from the null within DZ twins (OR=1.46, 95% CI [1.11, 1.93]), it became non-significant within MZ twins (OR=0.89, 95% CI [0.59, 1.33] (Fig. 2). Thus, within identical twin pairs, regardless of which twin had higher scores on the general pain factor, both twins had a roughly equal risk of later suicidal behavior.

With regard to the specific factors, the somatic symptoms factor was associated with suicidal behavior (OR=1.46, 95% CI[0.89, 2.39]) within all twins. However, akin to the general pain factor, this within-pair association appeared to be entirely driven by the DZ twins (OR=1.56, 95% CI [0.89, 2.75]), as the association was nearly null within MZ twins (OR=1.02, 95% CI [0.49, 2.11]).

### Sensitivity analyses

The results based on the sensitivity analyses were largely consistent with the main results (Additional file 1: Fig. S2-S5).

## Discussions

This study used nationwide Swedish twin data to examine the longitudinal association between self-reported chronic pain conditions and clinically assessed suicidal behavior. We observed that all chronic pain conditions were associated with increased risk of later suicidal behavior. However, after controlling the other pain conditions in a multiple regression, few associations remained statistically significant. This pattern of univariate and multivariate results is consistent with previous research that has had difficulty determining whether risk of suicidal behavior is specific to one or more pain conditions [8, 10, 67], and suggests the possibility that a shared liability underlying the chronic pain conditions explains their observed associations with risk of suicidal behavior. We explicitly modeled this underlying liability by examining general and condition-specific processes. We found that the general factor of pain was significantly phenotypically associated with suicidal behavior, as was the specific factor representing risk for somatic symptoms (chronic fatigue, irritable bowel, headache, and bladder pain).

Our phenotypic results are consistent with previous studies demonstrating that non-cancer pain conditions and pain intensity are associated with an increased risk of suicidal ideation, attempts, and deaths, even when adjusted for measured confounders (e.g., mental health conditions) [68–72]. However, given the difficulty of adequately addressing confounding with measured covariates [66], the threat of bias from residual confounding has made it difficult to understand the causal processes underlying these associations [37]. To expand upon previous studies of pain and suicide, we used a co-twin control comparison to adjust for unmeasured confounding [44, 73]. The co-twin control design is potentially powerful in that it adjusts for all factors in the early environment or upbringing that make twins similar, without requiring them to be measured or known. Moreover, relative to the direct associations between pain diagnoses and suicide, we evaluated the associations via latent pain factors that should limit measurement error. With this arguably stronger study design, we found quite different results: A general liability of pain was associated with suicidal behavior within DZ twins. However, this liability was null within the MZ twins, which represented our strongest adjustment for familial confounding. We found a similar pattern for the somatic symptom-specific factor. Thus, our result suggested that observed associations between pain conditions and suicidal behavior might be explained at least in part by shared genetics rather than a phenotypic (causal) influence of general or specific pain liability on later suicidal behavior.

These findings raise the possibility that treating pain itself may not reduce risk of later suicide. Importantly, beyond this non-causal interpretation, the attenuation could occur due to two other reasons. First, genetics could influence phenotypic pain, which in turn influences suicidal behavior (i.e., pain mediates the genetics-suicide association). We believe that this first alternate explanation might be unlikely because the co-twin model does not adjust for non-shared environmental factors, and any remaining twin pair differences then ought to remain associated with suicide. Second, the null association could also be explained by low statistical power or pain measurement error (although we note that we limited measurement error via the latent factor model in our study). Regardless, we did replicate prior findings of greater risk of suicidal behavior among those with pain conditions. Suicide screening in this population and, when relevant, suicide-specific interventions would clearly be recommended, even if the pain conditions themselves may not contribute to the elevated risk of suicidal behavior.

As suggested by the difference between the DZ and MZ co-twin control associations, shared genetic factors, in particular, might confound the causal influence of pain to suicide. We believe these explanations are plausible, as there are genetic influences on general pain liability as well as on suicide [35, 36, 41, 42]. One possibility is that genetic influences on the endogenous opioid system may at least in part underlie both chronic pain and suicidal behavior [28]. Moreover, ample research demonstrates shared genetic influences on chronic pain and psychopathology other than suicide [34, 36, 74, 75].

Unlike previous studies of chronic pain conditions that have modeled their comorbidity via multiple regression, we used a novel bifactor analysis to summarize their intercorrelations into one general factor of pain and two specific latent factors (somatic symptoms, neck-shoulder pain). Comorbidity among chronic pain conditions has been proposed to be explained by shared genetic, neurobiologic, and psychosocial factors [35, 36, 76]. This implies that understanding associations of specific pain syndromes with outcomes like suicidal behavior requires considering general processes as well. The bifactor approach offers a solution to this problem. Our results suggest that there may be a general factor that accounts for meaningful variance across major pain syndromes, which might help clinicians inform treatment management plans and predict pain prognosis.

In addition to the general pain factor, we extracted two factors representing covariance among specific conditions. The somatic symptoms factor could reflect a second etiologic contribution to symptoms other than musculoskeletal pain. The second specific factor corresponded to neck and shoulder pain, perhaps capturing pain due to injury or other causes at these locations after extracting the general factor of pain. Neck- and shoulder-specific pain did not appear to be associated with risk of suicidal behavior.

Our results show that patients suffering from chronic pain are at greater risk of suicide, even if the chronic pain itself may not substantially contribute to that risk. Future research may benefit from involving other specific chronic pain conditions (e.g., temporomandibular disorder, vulvodynia, migraine or other specific chronic headache conditions), including measures of more chronic and/or severe pain, identifying risk factors for suicide among patients with chronic pain, and evaluating interventions to reduce suicide among patients with chronic pain.

### Limitations

First, we did not involve psychopathology in our model, although mental health conditions are associated with both chronic pain and suicidal behavior. Prior studies of the pain-suicide association have included mental health conditions as statistical covariates [68], but doing so may pose interpretive challenges given that they may play multiple roles (e.g., mediator versus confounder) in the causal pathway from chronic pain conditions to the suicidal behavior. To avoid this difficulty, the present study represents the first, to our knowledge, application of a co-twin control design to study the pain-suicide association. This approach adjusted for shared familial confounding by design (i.e., without requiring measured statistical covariates). There could still be within-pair confounding from psychopathology or other factors, although such confounding would in all likelihood suggest that the attenuated and not statistically significant results observed here actually overestimated any causal effect of the chronic pain conditions. Second, we analyzed a population-based cohort of Swedish twins with loss to follow-up of only those twins who died or emigrated. However, selection bias is possible due to the rates of participation in the STAGE survey and completion of the pain measures. Third, our outcome consisted of clinically recognized suicidal behavior in nationwide register-based data, although undiagnosed suicidal behavior likely resulted in at least some misclassification. We included injuries of undetermined intent to attempt to address this limitation, but some suicidal behavior is not clinically recognized and thus would not be available in the register data. We additionally combined diagnosed suicidal behavior with death by suicide given the rarity of the latter outcome, although there may be etiologic differences between suicide attempts and deaths [77]. Fourth, the greater control for genetic confounding in MZ (vs. DZ) twin comparisons is not the only potential explanation for differing zygosity results. Because of the greater concordance among MZ vs. DZ pairs, MZ comparisons can be susceptible to greater bias due to measurement error [66]. Moreover, zygosity group differences may also reflect simple sampling variation. Thus, our analysis cannot definitively demonstrate the source of the familial confounding. Understanding shared genetic or other etiologic pathways to chronic pain and suicide represents an important area for future research. Fifth, we did not explore opioid or other treatments in our study, although opioid treatment might increase the risk of suicide, whereas other pain treatments might be protective.

## Conclusions

Both general and somatic pain were independently associated with suicidal behavior. However, the associations attenuated and became non-significant within MZ twin pairs. Clinicians might benefit from measuring not only specific types of pain, but also pain comorbidity; however, our results raise the possibility that treating pain might not necessarily reduce future suicide behavior.

## Supplementary Information

**Additional file 1: Fig S1.** Bifactor confirmatory factor analysis model of pain scales. **Fig S2.** Associations between pain conditions and suicidal behaviors using univariate and multivariate logistic regression model, excluding participants with suicide diagnoses before the STAGE survey. **Fig S3.** Associations between pain conditions and suicidal behaviors using univariate and multivariate logistic regression model, excluding the undetermined intent diagnoses. **Fig S4.** Associations between pain conditions and suicidal behaviors using univariate and multivariate Cox regression model. **Fig S5.** Associations between pain conditions and suicidal behaviors using univariate and multivariate logistic regression model, including self-reported somatic disorders as covariates. **Table S1.** Frequency distribution of the nine pain conditions among MZ and DZ twins. **Table S2.** Frequency distribution of the nine pain conditions among suicidal behaviors. **Table S3.** Standardized factor loadings from the confirmatory bifactor model based on 9 pain scales. **Supplemental Method.** Details of chronic pain items and scales.

## Data Availability

The Public Access to Information and Secrecy Act in Sweden prohibits us from making individual-level data publicly available. Researchers who are interested in replicating our work can apply for individual-level data through Statistics Sweden at: https://www.scb.se/en/services/guidancefor-researchers-and-universities/.
