# Supplementary material for: Chronic pain conditions and risk of suicidal behavior: a 10-year longitudinal co-twin control study
Source: BMC Med. 2023 Jan 5;21:9. doi: 10.1186/s12916-022-02703-8 (PMC9814420; doi:10.1186/s12916-022-02703-8)
Supplement: Supplementary file 1 — Additional file 1: Fig S1. Bifactor confirmatory factor analysis model of pain scales. Fig S2. Associations between pain conditions and suicidal behaviors using univariate and multivariate logistic regression model, excluding participants with suicide diagnoses before the STAGE survey. Fig S3. Associations between pain conditions and suicidal behaviors using univariate and multivariate logistic regression model, excluding the undetermined intent diagnoses. Fig S4. Associations between pain conditions and suicidal behaviors using univariate and multivariate Cox regression model. Fig S5. Associations between pain conditions and suicidal behaviors using univariate and multivariate logistic regression model, including self-reported somatic disorders as covariates. Table S1. Frequency distribution of the nine pain conditions among MZ and DZ twins. Table S2. Frequency distribution of the nine pain conditions among suicidal behaviors. Table S3. Standardized factor loadings from the confirmatory bifactor model based on 9 pain scales. Supplemental Method. Details of chronic pain items and scales. [file 12916_2022_2703_MOESM1_ESM.docx]

**Figure S1.** Bifactor confirmatory factor analysis model of pain scales.


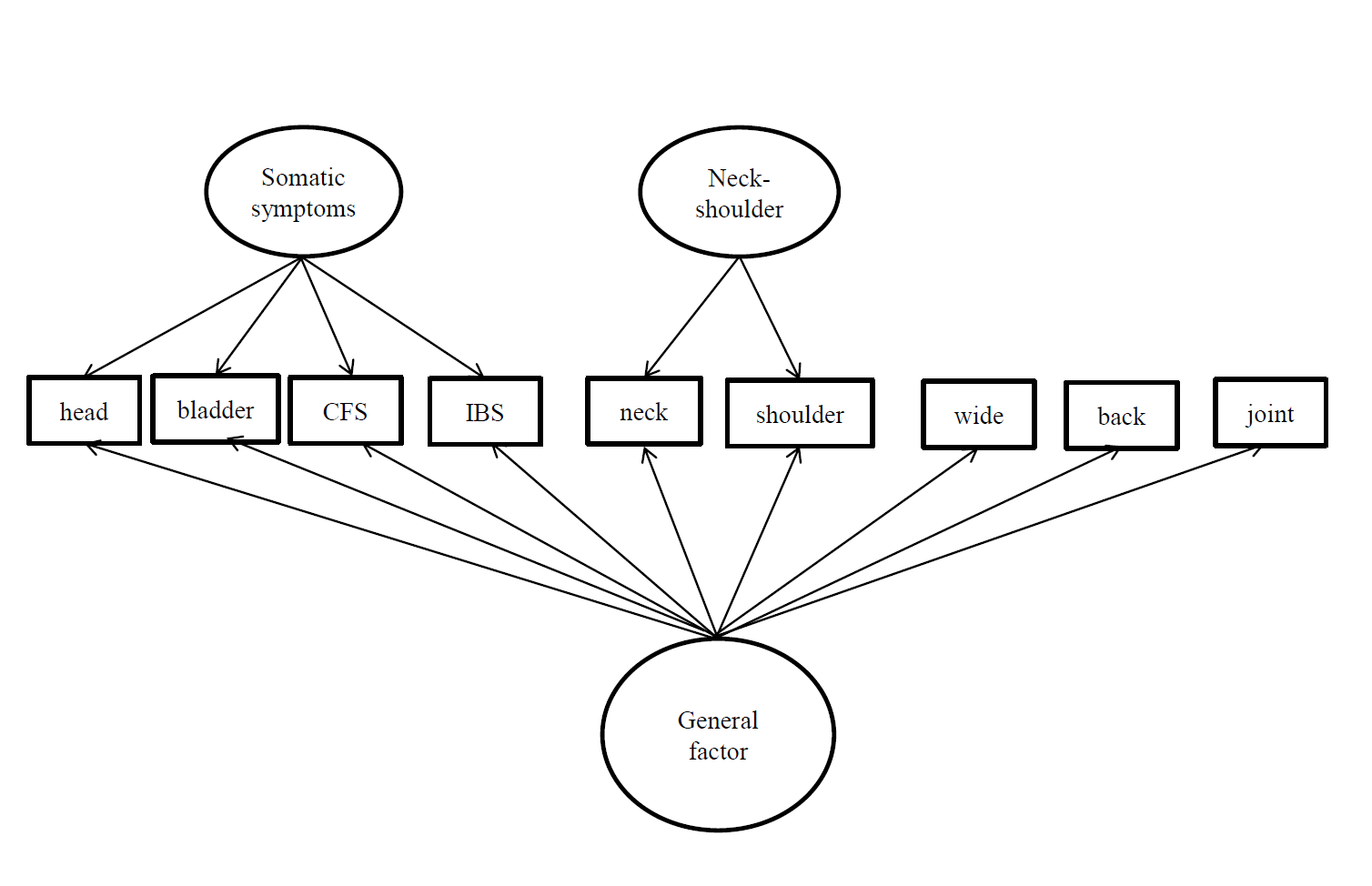


**Note:** Path diagram of the general factor model based on pain scales. The model consisted of a latent general factor of pain and two specific latent factors reflecting pain dimensions of somatic symptoms and neck-shoulder pain. All specific latent factors were correlated with each other. Variance for all latent factors were fixed at 1. Measured variables are depicted as squares, and include headache (head), bladder pain (bladder), chronic fatigue syndrome (CFS), irritable bowel syndrome (IBS), neck pain (neck), shoulder pain (shoulder), chronic widespread pain (wide), lower back pain (back), and joint pain (joint).

**Figure S2.** Associations between pain conditions and suicidal behaviors using univariate and multivariate logistic regression model, excluding participants with suicide diagnoses before the STAGE survey.


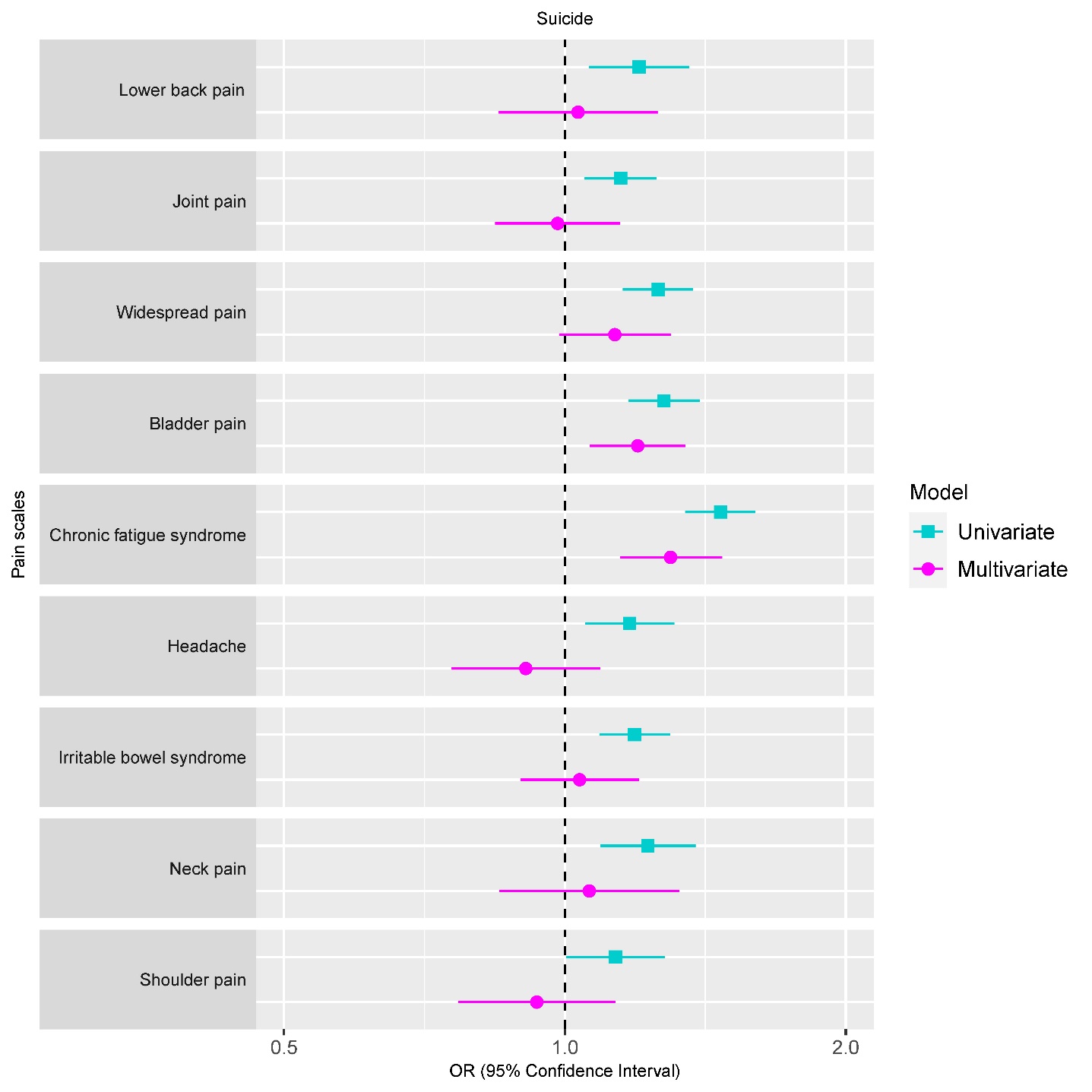


Note: Models were adjusted for age, sex and cancer.

**Figure S3.** Associations between pain conditions and suicidal behaviors using univariate and multivariate logistic regression model, excluding the undetermined intent diagnoses.


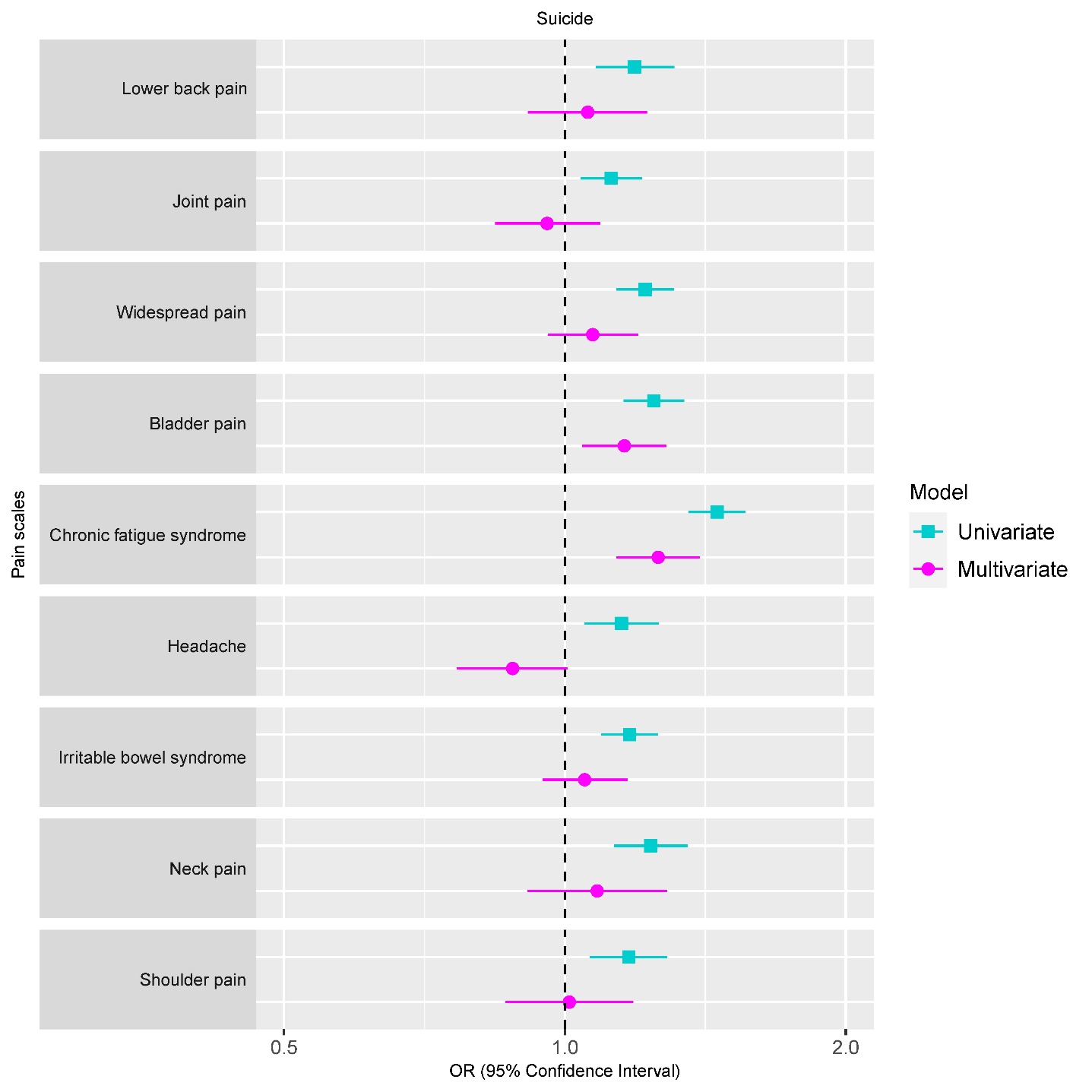


Note: Models were adjusted for age, sex and cancer.

**Figure S4.** Associations between pain conditions and suicidal behaviors using univariate and multivariate Cox regression model.


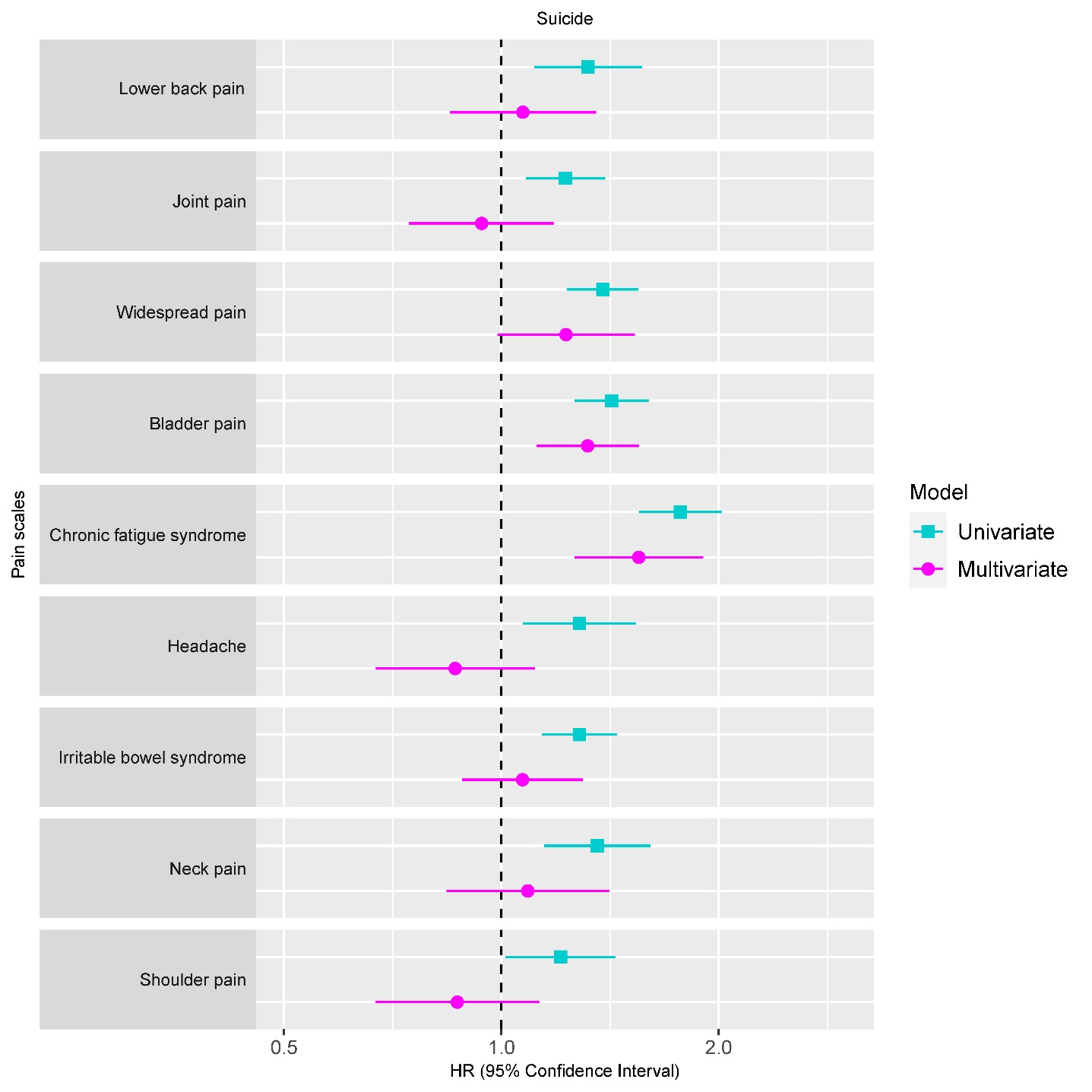


Note: Models were adjusted for age, sex and cancer.

**Figure S5.** Associations between pain conditions and suicidal behaviors using univariate and multivariate logistic regression model, including self-reported somatic disorders as covariates.


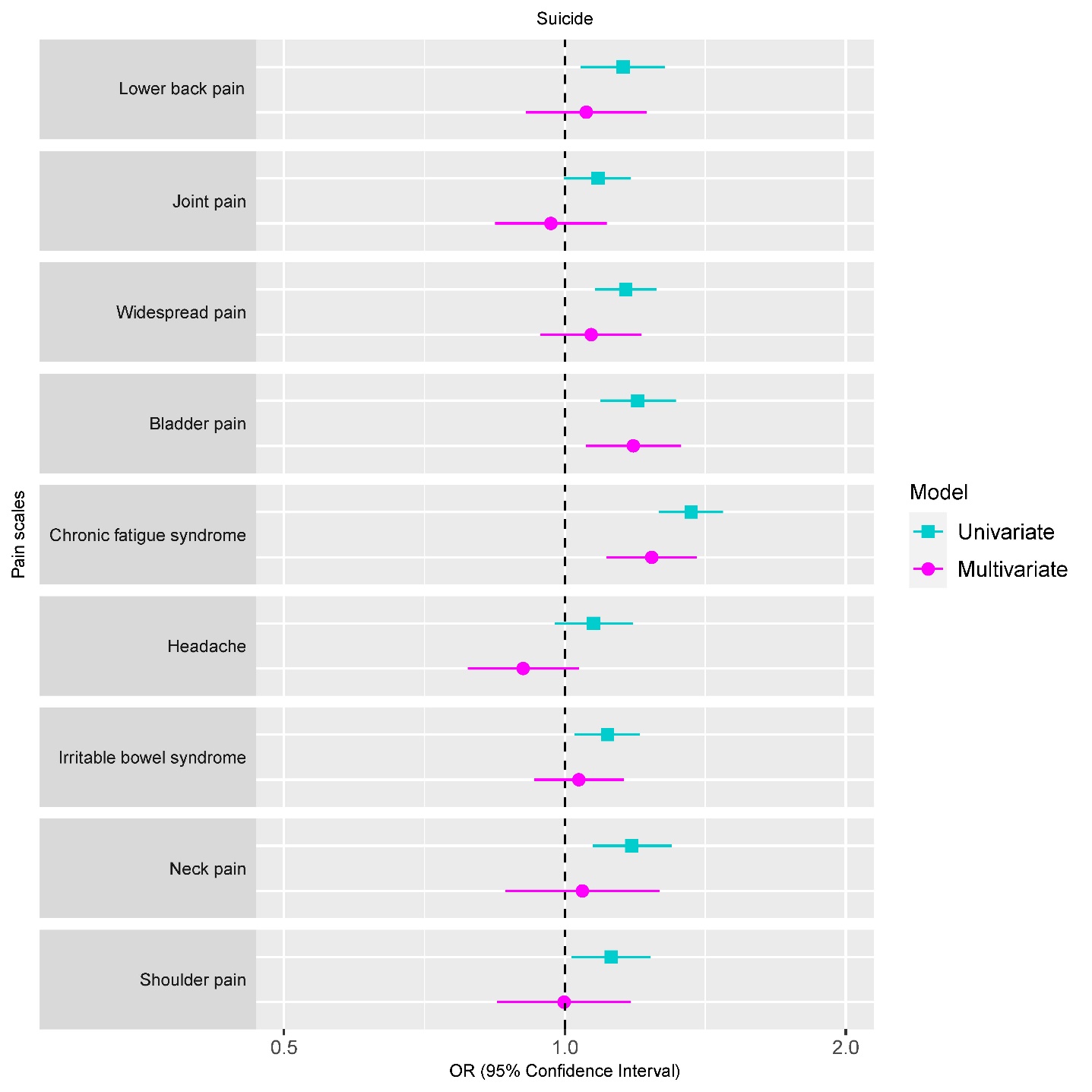


Note: Models were adjusted for age, sex, cancer, chronic bronchitis, Crohn's disease, diabetes, glandular disease (including everything but goiter), irregular cardiac rhythm/atrial fibrillation, liver disease (for example, cirrhosis), multiple sclerosis, goiter, ulcerative colitis, recurring urinary tract problems, intestinal (duodenal) ulcer, stomach ulcer.

**Table S1.** Frequency distribution of the nine pain conditions among MZ and DZ twins.

| **Chronic pain conditions**  **(1=yes, 0=no, NA=missing)** | **MZ twins**  **(N=7126)** | **DZ twins**  **(N=10 022)** | **Total**  **(N=17 148)** |
| --- | --- | --- | --- |
| Chronic Widespread Pain |  |  |  |
| 0 | 4936 | 6733 | 11669 |
| 1 | 689 | 1013 | 1702 |
| NA | 1501 | 2276 | 3777 |
| Joint Pain |  |  |  |
| 0 | 6055 | 8257 | 14312 |
| 1 | 398 | 576 | 974 |
| NA | 673 | 1189 | 1862 |
| Lower Back pain |  |  |  |
| 0 | 3628 | 5065 | 8693 |
| 1 | 1150 | 1767 | 2917 |
| NA | 2348 | 3190 | 5538 |
| Neck Pain |  |  |  |
| 0 | 3656 | 5175 | 8831 |
| 1 | 1143 | 1662 | 2805 |
| NA | 2327 | 3185 | 5512 |
| Shoulder Pain |  |  |  |
| 0 | 3874 | 5428 | 9302 |
| 1 | 923 | 1411 | 2334 |
| NA | 2329 | 3183 | 5512 |
| Irritable Bowel Syndrome |  |  |  |
| 0 | 5843 | 8101 | 13944 |
| 1 | 828 | 1077 | 1905 |
| NA | 455 | 844 | 1299 |
| Headache |  |  |  |
| 0 | 2021 | 2869 | 4890 |
| 1 | 4510 | 6019 | 10529 |
| NA | 595 | 1134 | 1729 |
| Bladder Pain |  |  |  |
| 0 | 4645 | 6612 | 11257 |
| 1 | 1458 | 1848 | 3306 |
| NA | 1023 | 1562 | 2585 |
| Chronic Fatigue Syndrome |  |  |  |
| 0 | 4835 | 6528 | 11363 |
| 1 | 1664 | 2333 | 3997 |
| NA | 627 | 1161 | 1788 |

**Table S2.** Frequency distribution of the nine pain conditions among suicidal behaviors.

| **Chronic pain conditions**  **(1=yes, 0=no, NA=missing)** | **Suicidal behaviors**  **(No=16 878)** | **Suicidal behaviors**  **(Yes=270)** | **Total**  **(N=17 148)** |
| --- | --- | --- | --- |
| Chronic Widespread Pain |  |  |  |
| 0 | 11505 | 164 | 11669 |
| 1 | 1649 | 53 | 1702 |
| NA | 3724 | 53 | 3777 |
| Joint Pain |  |  |  |
| 0 | 14101 | 211 | 14312 |
| 1 | 947 | 27 | 974 |
| NA | 1830 | 32 | 1862 |
| Lower Back pain |  |  |  |
| 0 | 8576 | 117 | 8693 |
| 1 | 2853 | 64 | 2917 |
| NA | 5449 | 89 | 5538 |
| Neck Pain |  |  |  |
| 0 | 8712 | 119 | 8831 |
| 1 | 2742 | 63 | 2805 |
| NA | 5424 | 88 | 5512 |
| Shoulder Pain |  |  |  |
| 0 | 9172 | 130 | 9302 |
| 1 | 2283 | 51 | 2334 |
| NA | 5423 | 89 | 5512 |
| Irritable Bowel Syndrome |  |  |  |
| 0 | 13761 | 183 | 13944 |
| 1 | 1853 | 52 | 1905 |
| NA | 1264 | 35 | 1299 |
| Headache |  |  |  |
| 0 | 4827 | 63 | 4890 |
| 1 | 10347 | 182 | 10529 |
| NA | 1704 | 25 | 1729 |
| Bladder Pain |  |  |  |
| 0 | 11127 | 130 | 11257 |
| 1 | 3215 | 91 | 3306 |
| NA | 2536 | 49 | 2585 |
| Chronic Fatigue Syndrome |  |  |  |
| 0 | 11251 | 112 | 11363 |
| 1 | 3871 | 126 | 3997 |
| NA | 1756 | 32 | 1788 |

**Table S3.** Standardized factor loadings from the confirmatory bifactor model based on 9 pain scales.

| Scale | General factor | Somatic symptoms | Neck-shoulder pain |
| --- | --- | --- | --- |
| Lower back pain | 0.65 | 0 | 0 |
| Joint pain | 0.59 | 0 | 0 |
| Widespread pain | 0.99 | 0 | 0 |
| Bladder pain | 0.32 | 0.37 | 0 |
| Chronic fatigue syndrome | 0.52 | 0.33 | 0 |
| Headache | 0.40 | 0.33 | 0 |
| Irritable bowel syndrome | 0.36 | 0.49 | 0 |
| Neck pain | 0.70 | 0 | 0.71 |
| Shoulder pain | 0.66 | 0 | 0.34 |

**Supplemental Method**

**Pain Conditions**

We assessed nine chronic pain conditions using self-reports provided in the Study of Twin Adults: Genes and Environment (STAGE). Where possible, we applied operationalizations that have demonstrated validity in prior studies. To maximize variation, we used counts of symptoms or criteria rather than dichotomous syndrome categories where relevant.

***Chronic Widespread Pain***

Chronic widespread pain items were administered to those endorsing pain in at least two parts of the body within the past six months (i.e., shoulders/shoulder blades, neck, upper or lower back, elbows, hip, knees and feet/ankles). We created a five-level variable using the following four criteria: general pain within the last three months, continuous pain within the last three months, pain in both upper and lower body, and pain in both the left and right sides of the body. Participants with pain in fewer than two body parts in the past six months were assumed to have no chronic widespread pain. This operational definition is similar to that used in other Swedish Twin Registry (STR) studies [30, 34, 47, 48] and is based on the 1990 fibromyalgia criteria proposed by the American College of Rheumatology (ACR) without tender points [49].

***Joint Pain***

We assessed joint pain with a dichotomous indicator of joint pain lasting for more than six months, which is similar to the index of prolonged joint pain used in prior STR studies, albeit with a greater duration (six months rather than four weeks; [34]).

***Lower Back Pain, Neck Pain, and Shoulder Pain***

We created a four-level variable for lower back pain using three criteria (pain presence, intensity, and disability) following Nyman et al.'s ([50, 51]) operational definition in STAGE. Participants responded to a first criterion regarding whether they had experienced lower back pain within the last six months (accompanied by a drawing of body regions). Those who endorsed lower back pain were then asked three questions on 0-10 scales regarding intensity (at the moment, worst pain during the past six months, and average pain during the past six months) and regarding disability (in everyday activities, social life, and work). Intensity and disability were assumed to be absent among those without any past-six-month pain. We considered participants as meeting the intensity criterion with an average intensity score of 3 or greater and as meeting the disability criterion with an average score of 1 or greater. Neck pain and shoulder pain were each assessed using the same algorithm with corresponding items.

***Irritable Bowel Syndrome (IBS)***

We created a seven-level variable for IBS using an operational definition following Svedberg et al. [52] , which closely aligned with the Rome criteria [53]. Participants who reported recurrent abdominal discomfort for at least seven days per month responded to questions about six criteria: lower abdominal pain, bloating, constipation, diarrhea, abdominal pain when feces become looser and defecation more frequent, and alleviation of abdominal pain after defecation. Participants who did not endorse discomfort lasting at least seven days per month were assumed to have met no IBS criteria. These IBS criteria have been used in previous research as well [47, 48].

***Headache***

We divided four headache items into two categories to create a three-level variable (i.e., no headache, non-recurrent headache, and recurrent headache). The first category assessed the occurrence of headaches ever and within the past year without a specific cause (i.e., fever, hangover, or some other medical condition). The second category assessed the occurrence of recurrent headaches or daily or almost daily headaches. Endorsing at least one of the questions in the first category without endorsing either in the second indicated the existence of non-recurrent headache, whereas endorsing at least one of the questions in the second category indicated the existence of recurrent headache. These criteria closely follow those used in several other STR studies of recurrent headache [34, 47, 54] .

***Bladder Pain***

Following Altman et al.'s [55] STAGE bladder pain syndrome classification, we included four symptoms to create a five-level variable for bladder pain. We considered bladder pain to be present if participants endorsed lifetime pain/pressure/discomfort and rated it as greater than 0 on a 0-10 scale. Among those experiencing bladder pain, we assessed duration (at least 1 month) and relief of discomfort after urination. These symptoms were assumed to be absent among those not endorsing bladder pain. Finally, we assessed frequent (i.e., >1 per night) nocturnal urination.

***Chronic Fatigue Syndrome (CFS)***

We created a four-level variable for chronic fatigue (without ancillary symptoms such as muscle pain or headache) using three criteria. Our approach closely followed an STR instrument reported by Evengård et al. [56] , which itself aligns with the CDC conceptual framework for CFS [57] and has been used in other studies [34, 47, 48] . The included criteria were fatigue (i.e., feeling abnormally tired), six-month fatigue duration, and impairment (in life, work, or social life), with the latter two assumed to be absent among those not endorsing fatigue.
